# General SIR model for visible and hidden epidemic dynamics

**DOI:** 10.3389/frai.2025.1559880

**Published:** 2025-02-24

**Authors:** Igor Nesteruk

**Affiliations:** Institute of Hydromechanics, National Academy of Sciences of Ukraine, Kyiv, Ukraine

**Keywords:** mathematical modeling of infection diseases, SIR model, parameter identification, pertussis epidemic in England, hidden epidemic dynamics

## Abstract

To simulate hidden epidemic dynamics connected with asymptomatic and unregistered patients, a new general SIR model was proposed. For some cases, the analytical solutions of the set of 5 differential equations were found, which allow simplifying the parameter identification procedure. Two waves of the pertussis epidemic in England in 2023 and 2024 were simulated with the assumption of zero hidden cases. The accumulated and daily numbers of cases and the duration of the second wave were predicted with rather high accuracy. If the trend will not change, the monthly figure of 9 new pertussis cases (as it was in January–February 2023) can be achieved only in May 2025. The proposed approach can be recommended for both simulations and predictions of different epidemics.

## 1 Introduction

Asymptomatic and unregistered cases are characteristic of almost all infectious diseases, in particular, SARS-CoV-2 (Mass coronavirus testing in Slovakia-a, 2020; Mass coronavirus testing in Slovakia-b, 2020; An experiment with mass testing for COVID-19 was conducted in Khmelnytsky, n.d.; Schreiber et al., 2023; Fowlkes et al., 2022; Shang et al., 2022) and pertussis (Craig et al., 2020) are no exception. The percentage of asymptomatic patients can be age dependent and lead to huge differences in registered numbers of cases for countries with young and old population (Davies et al., 2020; Nesteruk, 2024b; Nesteruk and Keeling, 2023). Some theoretical estimations of the visibility coefficient β—the ratio of real infections to the registered ones can be found in Nesteruk (2024b), Nesteruk (2021e), Nesteruk (2021c), and Nesteruk (2021d). In this study we will use the concepts of the classical SIR (susceptible-infectious-removed) model (Kermack and McKendrick, 1927; Weiss, 2013; Daley and Gani, 2005; Keeling and Rohani, 2008; Cherniha, 2020; Mohammadi et al., 2021; Nesteruk, 2021a), its generalization for simulations of different epidemic waves (Nesteruk, 2021a; Nesteruk, 2021b; Nesteruk, 2023b) and procedures of parameter identification (Nesteruk, 2023b; Nesteruk, 2017). Numerous improvements of SIR model (see, e.g., Hethcote, 2000; Nakamura et al., 2020; Britton, 2004; Pesco et al., 2014; Nesteruk, 2023a) do not take into account the visibility coefficient.

The obtained theoretical results will be applied for simulations of the pertussis (whooping cough) epidemic in England in 2023 and 2024 (Confirmed cases of pertussis in England by month, n.d.). This disease increases the risk of infant fatality and became a serious problem in many countries including the developed ones (Pesco et al., 2014; Confirmed cases of pertussis in England by month, n.d.). Numerical differentiation of the monthly numbers of new cases revealed two waves of the epidemic in England (before and after November 2023) (Nesteruk, 2024a). Due to the absence of necessary amount of observations, SIR simulations were performed in Nesteruk (2024a) only for the first wave. In this study we will use the new approach for simulation of both waves of the pertussis epidemic and compare the predictions with the recent statistical data.

## 2 Differential equations and initial conditions

For every epidemic wave *i*, let us divide the compartment of infectious persons *I(t)* (*t* is time) into visible (registered) and hidden (invisible/asymptomatic and unregistered) parts $I=I^{\left(v\right)}+I^{\left(h\right)}$ and suppose that these persons are appearing according to the visibility coefficient $\beta_{i}\geq 1$ and removing with rates $\rho_{i}^{\left(v\right)}I^{\left(v\right)}$ and $\rho_{i}^{\left(h\right)}I^{\left(h\right)}$. Then the general SIR model (Nesteruk, 2021a; Nesteruk, 2021b; Nesteruk, 2023b) takes the following form:


(1) $$\frac{dS}{dt}=-\alpha_{i}S\left(I^{\left(v\right)}+I^{\left(h\right)}\right)$$

(2) $$\frac{dI^{\left(v\right)}}{dt}=\frac{\alpha_{i}}{\beta_{i}}S\left(I^{\left(v\right)}+I^{\left(h\right)}\right)-\rho_{i}^{\left(v\right)}I^{\left(v\right)}$$

(3) $$\frac{dI^{\left(h\right)}}{dt}=\frac{\left(\beta_{i}-1\right)\alpha_{i}}{\beta_{i}}S\left(I^{\left(v\right)}+I^{\left(h\right)}\right)-\rho_{i}^{\left(h\right)}I^{\left(h\right)}$$

(4) $$\frac{dR^{\left(v\right)}}{dt}=\rho_{i}^{\left(v\right)}I^{\left(v\right)}$$

(5) $$\frac{dR^{\left(h\right)}}{dt}=\rho_{i}^{\left(h\right)}I^{\left(h\right)}$$

The compartment of removed persons *R(t)* is also divided into visible (registered) and hidden parts $R=R^{\left(v\right)}+R^{\left(h\right)}$. Infection and removal rates ($\alpha_{i}$, $\rho_{i}^{\left(v\right)}$, $\rho_{i}^{\left(h\right)}$) and the visibility coefficient $\beta_{i}$ are supposed to be constant for every epidemic wave, i.e., for the time periods: $t_{i}^{*}\leq t\leq t_{i+1}^{*},\;i=1,2,3,\ldots$. Summarizing Equations 1–5 yields zero value of the derivative $d\left(S+I^{\left(v\right)}+I^{\left(h\right)}+R^{\left(v\right)}+R^{\left(h\right)}\right)/dt$. Then the sum:


(6) $$N_{i}=S+I^{\left(v\right)}+I^{\left(h\right)}+R^{\left(v\right)}+R^{\left(h\right)}$$

must be constant for every epidemic wave. We will consider the value *N_i_* to be an unknown parameter of the model corresponding to the *i-th* wave, which is not equal to the known volume of population and must be estimated by observations. There is no need to assume that before the outbreak all people are susceptible, since many of them are protected by their immunity, distance, lockdowns, etc. Thus, we will not reduce the problem to a 4-dimensional one. It means that the solution can be obtained by numerical integration of the set of 5 differential Equations 1–5. Nevertheless, there are some separate cases, when analytical solutions are possible (see next Section).

Taking into account Equation 6, the initial conditions for the set of Equations 1–5 at the beginning of every epidemic wave $t_{i}^{*}$ can be written as follows:


(7) $$\begin{array}{l} I^{\left(v\right)}\left(t_{i}^{*}\right)=I_{vi},\;I^{\left(h\right)}\left(t_{i}^{*}\right)=I_{hi},\;R^{\left(v\right)}\left(t_{i}^{*}\right)=R_{vi},\;R^{\left(h\right)}\left(t_{i}^{*}\right)=\\ R_{hi}\;S\left(t_{i}^{*}\right)=N_{i}-I_{vi}-I_{hi}-R_{vi}-R_{hi} \end{array}$$

If at moment $t_{i}^{*}$ all previously infected persons are removed, we can take into account only cases starting to appear during *i-th* wave and use the initial conditions:


(8) $$I_{vi}=1,\;I_{hi}=\beta_{i}-1,\;R_{vi}=0,\;R_{hi}=0$$

## 3 Examples of analytical solutions

Let us introduce the functions corresponding to the accumulated numbers of visible and hidden cases:


(9) $$V^{\left(v\right)}=I^{\left(v\right)}+R^{\left(v\right)},\;V^{\left(h\right)}=I^{\left(h\right)}+R^{\left(h\right)}$$

Then it follows from Equations 2–5 that


(10) $$\frac{dV^{\left(v\right)}}{dt}=\frac{\alpha_{i}}{\beta_{i}}S\left(I^{\left(v\right)}+I^{\left(h\right)}\right),\frac{dV^{\left(h\right)}}{dt}=\frac{\left(\beta_{i}-1\right)\alpha_{i}}{\beta_{i}}S\left(I^{\left(v\right)}+I^{\left(h\right)}\right)$$

Dividing Equation 10 by Equation 1 yeilds:


(11) $$\frac{dV^{\left(v\right)}}{dS}=-\frac{1}{\beta_{i}},\;\frac{dV^{\left(h\right)}}{dS}=-\frac{\left(\beta_{i}-1\right)}{\beta_{i}}$$

and simple linear solutions taking into account initial Equation 7:


(12) $$V^{\left(v\right)}=-\frac{S}{\beta_{i}}+\frac{N_{i}-I_{vi}-I_{hi}-R_{vi}-R_{hi}}{\beta_{i}}+I_{vi}+R_{vi},$$

(13) $$V^{\left(h\right)}=-\frac{\left(\beta_{i}-1\right)S}{\beta_{i}}+\frac{\left(\beta_{i}-1\right)\left(N_{i}-I_{vi}-I_{hi}-R_{vi}-R_{hi}\right)}{\beta_{i}}+I_{hi}+R_{hi}\text{.}$$

Equations 12, 13 allow obtaining simple linear relationship:


$$V^{\left(h\right)}=\left(\beta_{i}-1\right)V^{\left(v\right)}-\left(\beta_{i}-1\right)\left(I_{vi}+R_{vi}\right)+I_{hi}+R_{hi}$$

which demonstrates that the ratio of total accumulated cases $V=V^{\left(v\right)}+V^{\left(h\right)}$ to the registered ones:


(14) $$\frac{V}{V^{\left(v\right)}}=\beta_{i}-\frac{\left(\beta_{i}-1\right)\left(I_{vi}+R_{vi}\right)-I_{hi}-R_{hi}}{V^{\left(v\right)}}$$

is not constant and equals $\beta_{i}$ only approximately at large $V^{\left(v\right)}$ numbers. Equation 14 limits the accuracy of the approach used in Nesteruk (2021e), Nesteruk (2021c), and Nesteruk (2021d).

Introducing


(15) $$I=I^{\left(v\right)}+I^{\left(h\right)},\;I^{*}=\rho_{i}^{\left(v\right)}I^{\left(v\right)}+\rho_{i}^{\left(h\right)}I^{\left(h\right)}$$

summarizing Equations 2, 3 and dividing by Equation 1 yield the following differential equation:


(16) $$\frac{dI}{dS}=-1+\frac{I^{*}}{\alpha_{i}\text{IS}}$$

In 3 separate cases:

-
$\rho_{i}^{\left(v\right)}=\rho_{i}^{\left(h\right)}=\rho_{i}$

- $I^{\left(v\right)}>>I^{\left(h\right)},\;I^{\left(v\right)}\approx I$, $\rho_{i}=\rho_{i}^{\left(v\right)}$
- $I^{\left(v\right)}<<I^{\left(h\right)},\;I^{\left(h\right)}\approx I$, $\rho_{i}=\rho_{i}^{\left(h\right)}$

Equation 16 simplifies and has an analitycal solution taking into account the initial Equation 7:


(17) $$\frac{dI}{dS}≅-1+\frac{\rho_{i}}{\alpha_{i}S},$$

(18) $$I≅-S+N_{i}-R_{vi}-R_{hi}+\frac{\rho_{i}}{\alpha_{i}}\ln\frac{S}{N_{i}-I_{vi}-I_{hi}-R_{vi}-R_{hi}}$$

Equations 17, 18 exact in the case (I) and approximate in cases (II) and (III).

Putting Equation 18 into Equation 1 and integration yield:


(19) $$F\left(S\right)≅\alpha_{i}\left(t-t_{i}^{*}\right),$$

(20) $$F\left(S\right)\text{≡}\int_{N_{i}-I_{vi}-I_{hi}-R_{vi}-R_{hi}}^{S}\frac{dU}{U\left(\begin{array}{l} U-N_{i}+R_{vi}+R_{hi}\\ -\frac{\rho_{i}}{\alpha_{i}}\ln\frac{U}{N_{i}-I_{vi}-I_{hi}-R_{vi}-R_{hi}} \end{array}\right)}$$

It follows from Equations 1, 2, 15 that:


(21) $$\frac{dI^{\left(v\right)}}{dS}=-\frac{1}{\beta_{i}}+\frac{\rho_{i}^{\left(v\right)}I^{\left(v\right)}}{\alpha_{i}SI},$$

Taking into account that


$$\frac{dF}{dS}≅-\frac{1}{SI}$$

(see Equations 18, 20), the solution of the non-homogenous linear Equation 21 satisfying the first initial Equation 7 can be written as follows:


(22) $$\begin{array}{c} I^{\left(v\right)}\left(S\right)≅I_{vi}\exp\left(-\frac{\rho_{i}^{\left(v\right)}F\left(S\right)}{\alpha_{i}}\right)-\frac{1}{\beta_{i}}\exp\left(-\frac{\rho_{i}^{\left(v\right)}F\left(S\right)}{\alpha_{i}}\right)\times \\ \int_{N_{i}-I_{vi}-I_{hi}-R_{vi}-R_{hi}}^{S}\exp\left(\frac{\rho_{i}^{\left(v\right)}F\left(U\right)}{\alpha_{i}}\right)dU \end{array}$$

With the use of Equations 9, 15 it is possible to express other functions as follows:


(23) $$\begin{array}{l} I^{\left(h\right)}\left(S\right)≅I\left(S\right)-I^{\left(v\right)}\left(S\right);\\ R^{\left(v\right)}\left(S\right)≅V^{\left(v\right)}\left(S\right)-I^{\left(v\right)}\left(S\right);\\ R^{\left(h\right)}\left(S\right)≅V^{\left(h\right)}\left(S\right)-I^{\left(h\right)}\left(S\right) \end{array}$$

Then for every value of S, all unknown functions can be calculated with the use of Equations 12, 13, 18, 22, 23. Corresponding moments of time can be found with the use of Equations 19, 20. Thus, Equations 12, 13, 18–20, 22, 23 yield an approximate analytical solution of the set of differential Equations 1–5 with the initial Equation 7. In the case (I) and when $I^{\left(v\right)}=I$, this solution is exact. For $I^{\left(v\right)}=I$, there is no need in Equations 22, 23 and corresponding formulas obtained in Nesteruk (2021a), Nesteruk (2021b), Nesteruk (2023b) are also valid.

## 4 Examples of parameter identifications and predictions

The analytical solution simplifies the procedure of identification of unknown parameters, since there is no need in numerical integration of differential Equations 1–5. It particular, having the set of accumulated cases $V_{j}^{\left(v\right)}$ registered at moments *t_j_*, we can calculate corresponding values *S_j_* with the use of the linear Equation 12 for any values of unknown constant parameters appearing in Equations 1–7. Then Equation 20 allows calculating values *F_j_ = F(S_j_)*. Due to the linear relationship (Equation 19 shows that there is a linear dependence between time and the function F (Equation 20), which depends on the accumulated numbers of cases), standard linear regression formulas (Draper and Smith, 1998) can be used to calculate the correlation coefficient *r* and values of parameters $\alpha_{i}$ and $t_{i}^{*}$. The optimal values of model parameters (providing the best fitting between the theoretical $V^{\left(v\right)}\left(t\right)$ curves and the results of observations $V_{j}^{\left(v\right)}$) correspond to the maximum value of the correlation coefficient *r*. Thus, the parameter identification problem can be reduced to the problem of searching the maximum of complicated but analytical function *r*. For $I^{\left(v\right)}=I$ (a completely visible epidemic), such approach was successfully used to simulate and predict the dynamics of mysterious children disease (Nesteruk, 2017), COVID-19 pandemic (Nesteruk, 2021a; Nesteruk, 2021b; Nesteruk, 2023b) and the pertussis epidemic in England (Nesteruk, 2024a).

Let us illustrate the parameter identification procedure for two waves of the pertussis epidemic in England in 2023 and 2024 discussed in (Nesteruk, 2024a). The accumulated confirmed numbers of cases ($V_{j}^{\left(v\right)}$) and corresponding moments of time *t_j_* are listed in Table 1 according to the official site of UK government [(Confirmed cases of pertussis in England by month, n.d.), version available on 10 August 2024, the last 4 figures were taken from 10 December 2024 version]. The values $V_{j}^{\left(v\right)}$ were used to calculate approximate daily numbers of new cases *dV/dt* at moments *t_j_* according to the Equation 1 from (Nesteruk, 2024a) (see the last column in Table 1).

**Table 1:** Accumulated numbers of confirmed pertussis cases in England in 2023 and 2024 and estimations of the average daily numbers of visible cases.

Months, starting with January 2023	Days, starting with 1 January 2023, *t_j_*	Accumulated numbers of visible cases, $V_{j}^{\left(v\right)}$, (Confirmed cases of pertussis in England by month, n.d.)	Calculated daily numbers of new visible cases at moments *t_j_ dV/dt*, equation 1 in Nesteruk (2024a)
1	31	9	–
2	59	18	0.35426
3	90	30	0.52688
4	120	50	0.86559
5	151	83	1.41559
6	181	136	2.04462
7	212	208	2.66129
8	243	301	3.20
9	273	403	3.34516
10	304	505	3.47849
11	334	615	5.75269
12	365	858	12.87096
13	396	1,413	24.79644
14	425	2,332	38.86096
15	456	3,759	58.23280
16	486	5,872	84.44247
17	517	8,924	89.67581
18	547	11,351	67.65968
19	578	13,038	44.27419
20	609	14,096	28.79785
21	639	14,800	19.94301
22	670	15,309	–

Since the general problem contains 10 unknown parameters, their identification needs high performance computing and applying AI methods even for the analytical solution Equations 12, 13, 18–20, 22, 23. When this solution is approximate, the optimal values of parameters will contain discrepancies, which can reduce the accuracy of predictions. For our example, let us take the case of exact solution $I^{\left(v\right)}=I\;\left(\beta =1\right)$ and assume that at the beginning of every new epidemic wave all infectious persons from the previous waves are removed. Then we can use initial Equation 8, perform simulations and then add cases accumulated at moments when the monthly numbers of visible cases started to increase. For every wave we will have only four unknown parameters *N_i_*, $\alpha_{i}$, $\rho_{i}^{\left(v\right)}$ and $t_{i}^{*}$. Due to linear relation 19 and using linear regression, only two of these parameters are independent.

The first and second epidemic waves were simulated with the use of $V_{j}^{\left(v\right)}$ and *t_j_* corresponding to *j* = 3–9 and *j* = 11–18, respectively. The optimal values of parameters (corresponding to the maximum of correlation coefficients *r* = 0.999721131761662 (0.999822556136920)) are:

Ni = 50739.992 (3657890.47292358);

$\alpha_{i}$ =1.50488802805402e-05 (2.11266650706657e-06) [day]^−1^;

$\rho_{i}^{\left(v\right)}$=0.752503596035381 (7.7088126606047) [day]^−1^;

$t_{i}^{*}$=89.1566573802040 (333.429529143536) days.

(figures in brackets correspond to the second wave). It should be noted that these optimal values are very different for the first and second waves and differ from the figures in Nesteruk (2024a) for the simulation of the first wave using observations with *j* = 1–10.

**Figure 1:**
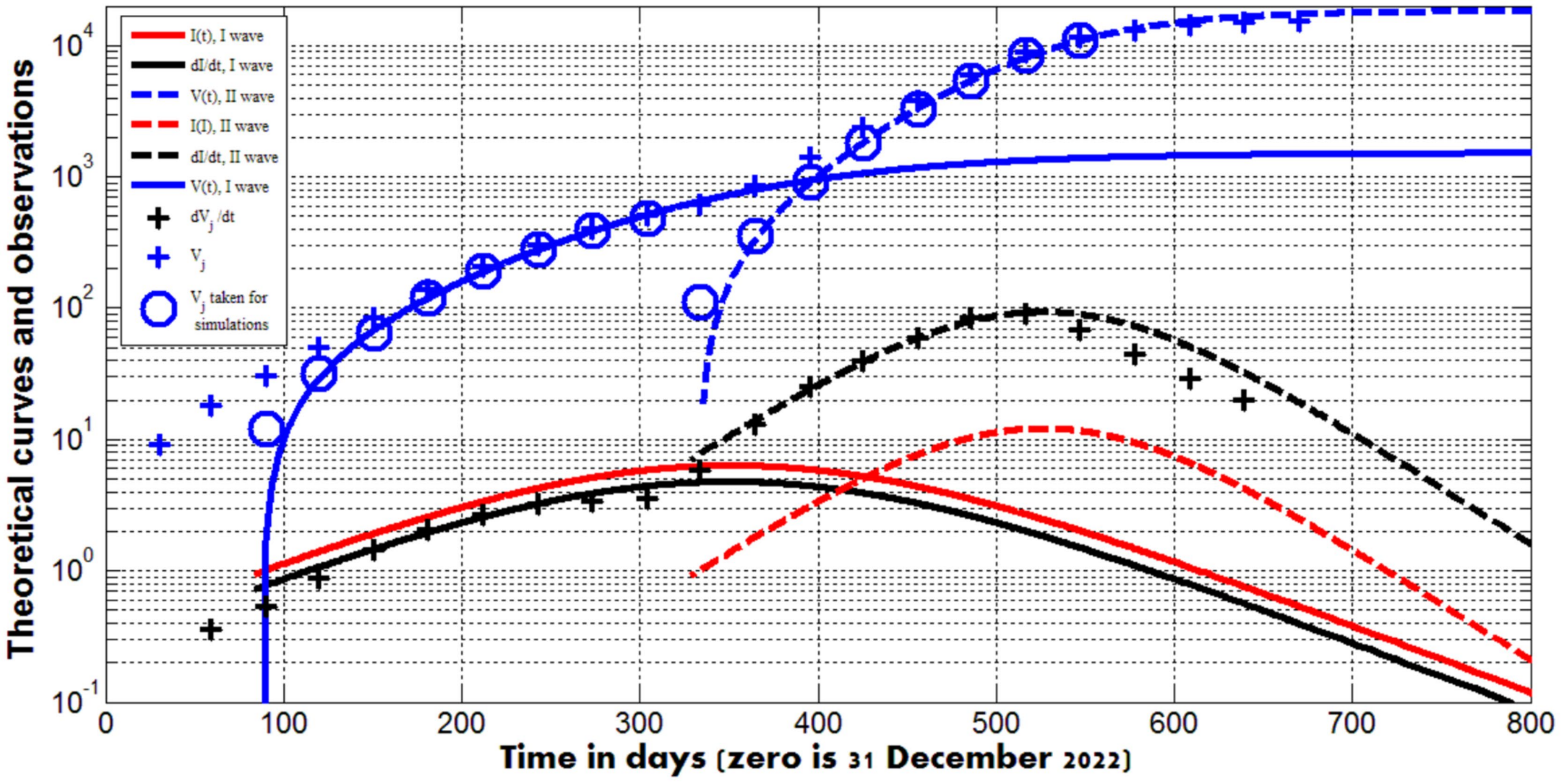
Accumulated numbers of visible pertussis cases (blue curves, the first Equation 9); the average daily numbers of new cases (black curves, the first Equation 10); numbers of infectious persons (red curves, Equation 18). “Circles” represent the confirmed numbers of cases $V_{j}^{\left(v\right)}$ taken for identification of parameters of the first (*j* = 3–10) and second (*j* = 11–18) waves; blue “crosses” – all confirmed numbers of cases $V_{j}^{\left(v\right)}$ listed in Table 1; black “crosses” – results of calculations of approximate daily numbers of new cases at moments *t_j_* listed in the last column of Table 1.

Using these optimal values in the analytical solution Equations 10, 12, 18–20, 22, 23 yielded the theoretical curves shown in Figure 1 (solid and dashed for the first and the second wave, respectively). The predicted values (see blue and black “crosses” for *j* = 19–22, July–October 2024) are in good agreement with the theoretical blue and black curves. To estimate the accuracy of 4-month prediction, let us take the accumulated number of visible cases $V_{22}^{\left(v\right)}$ =15,309 corresponding to the end of October 2024 (see Table 1) and compare with the theoretical value $V^{\left(v\right)}{|}_{t=670}$= 17,430 corresponding to the blue dashed line in Figure 1. After adding 505 cases accumulated at *t_10_* and extracted for the simulation of the second wave (compare blue “crosses” and “circles” in Figure 1), we obtain the accuracy (17,935-15,309)/15,309 around 17%. Since the final number of visible cases decreases with the increase of the visibility coefficient (see the next Section), we can expect to obtain a lower theoretical value $V^{\left(v\right)}{|}_{t=670}$ and better accuracy after the real visibility coefficient will be calculated and taken into account. The accuracy of 17% is comparable with the long-time predictions for the first waves of the COVID-19 in different countries (Nesteruk, 2021a) and even for the case β=1 is likely to allow healthcare professionals to develop the right strategy. The average daily numbers of new cases will be less than 1.0 only in March 2025. If trend will not change, the monthly figure of 9 new cases (as it was before starting the first wave, see Table 1) can be achieved only in May 2025.

## 5 Examples of exact solutions at different values of the visibility coefficient

The use of initial Equation 8 allows reducing the numbers of unknown parameters by 4. Then, Equations 12, 14 yield


(24) $$V^{\left(v\right)}=\frac{N_{i}-S}{\beta_{i}},$$

(25) $$V=\beta_{i}V^{\left(v\right)}$$

According to Equation 25 the real accumulated numbers of new cases *V* are $\beta_{i}$ times higher than visible figures $V^{\left(v\right)}$ registered during the fixed epidemic wave, if all infectious patients were removed before this wave started.

In the case (I), i.e., equal removing rates for visible and hidden patients $\rho_{i}^{\left(v\right)}=\rho_{i}^{\left(h\right)}=\rho_{i}$, another simplification can be obtained with the use of Equation 18:


(26) $$I=-S+N_{i}+\frac{\rho_{i}}{\alpha_{i}}\ln\frac{S}{N_{i}-\beta_{i}}$$

Assuming that spreading the infection stops when the real number of infectious *I* (visible and hidden) is less than 1.0, the corresponding final number of susceptible *S_f_* can be obtained as a solution of the non-linear equation:


(27) $$S_{f}+1=N_{i}+\frac{\rho_{i}}{\alpha_{i}}\ln\frac{S_{f}}{N_{i}-\beta_{i}}$$

following from Equation 26 and allowing us to calculate the corresponding final accumulated numbers of visible and total cases with the use of Equations 24, 25.

**Figure 2:**
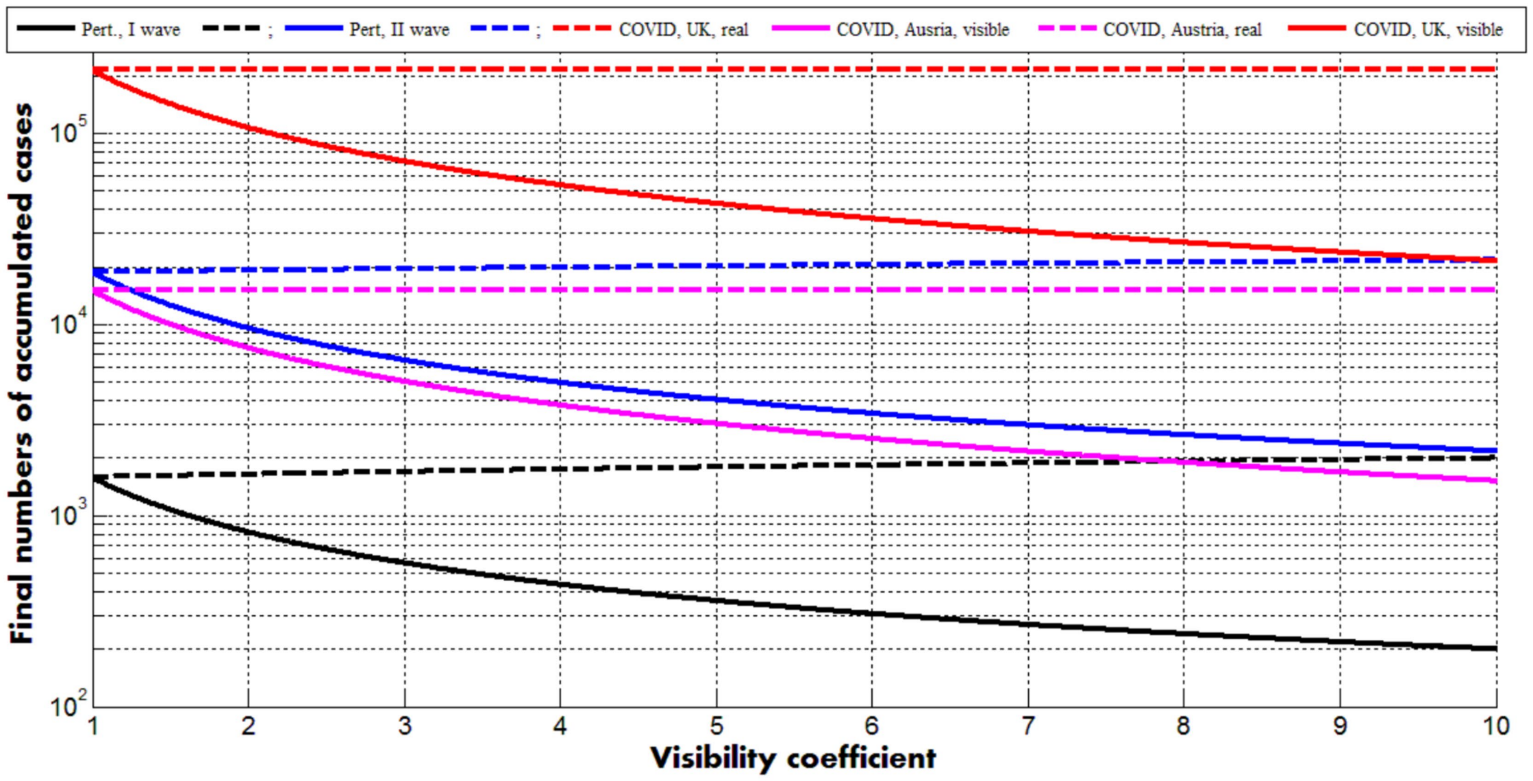
Solid curves represent final numbers of visible (registered) cases (Equations 24, 27) dashed ones–final numbers of all cases (registered and unregistered, Equations 25, 27). Back and blue lines correspond to the optimal values of parameters (listed in previous Section) for the first and second pertussis waves in England, respectively. Magenta and red curves show the results for the first COVID-19 waves in Austria and the UK (Nesteruk, 2021a), respectively.

Figure 2 represents the results of calculations for the optimal values of parameters corresponding to two pertussis waves in England (see previous Section) and the first COVID-19 pandemic waves in Austria and the UK (in brackets), (Nesteruk, 2021a):

*N_i_* = 75176.032 (479782.4);

$\alpha_{i}$ =1.924971386379e-05 (9.1371956639e-07) [day]^−1^;

$\rho_{i}^{\left(v\right)}$=1.29635017900866 (0.330545378991741) [day]^−1^.

Dashed lines demonstrate that the final accumulated numbers of all cases (Equations 25, 26) very slightly depend on the visibility coefficient. The final accumulated numbers of visible cases (Equations 24, 26) diminish with the increase of $\beta_{i}$ (see solid curves). The values of other parameters are fixed and correspond to the case $\beta_{i}$=1. The values $V_{j}^{\left(v\right)}$ for *j* = 20, 21, 22, which are smaller then theoretical prediction for the second pertussis wave (compare blue “crosses” and the blue dashed line in Figure 1), reflect reducing the final value of $V^{\left(v\right)}$ for $\beta_{i}$>1. Nevertheless, good estimations of the visibility coefficient can be obtained only with the use of all parameters. Since removing rates can be different for symptomatic and asymptomatic patients, a general parameter identification procedure needs a numerical solution of the set of differential Equations 1–5 and huge numbers of calculations, which can be performed only with the use of high performance computing and AI methods. The full parameter sensitivity analysis will be considered in future research.

With the use of Equations 24, 10, 26 can be rewritten as follows:


(28) $$I=\beta_{i}V^{\left(v\right)}+\frac{\rho_{i}}{\alpha_{i}}\ln\frac{N_{i}-\beta_{i}V^{\left(v\right)}}{N_{i}-\beta_{i}},$$

(29) $$\frac{dV^{\left(v\right)}}{dt}=\frac{\alpha_{i}}{\beta_{i}}\left(N_{i}-\beta_{i}V^{\left(v\right)}\right)I,\;\frac{dV}{dt}=\alpha_{i}\left(N_{i}-\beta_{i}V^{\left(v\right)}\right)I$$

**Figure 3:**
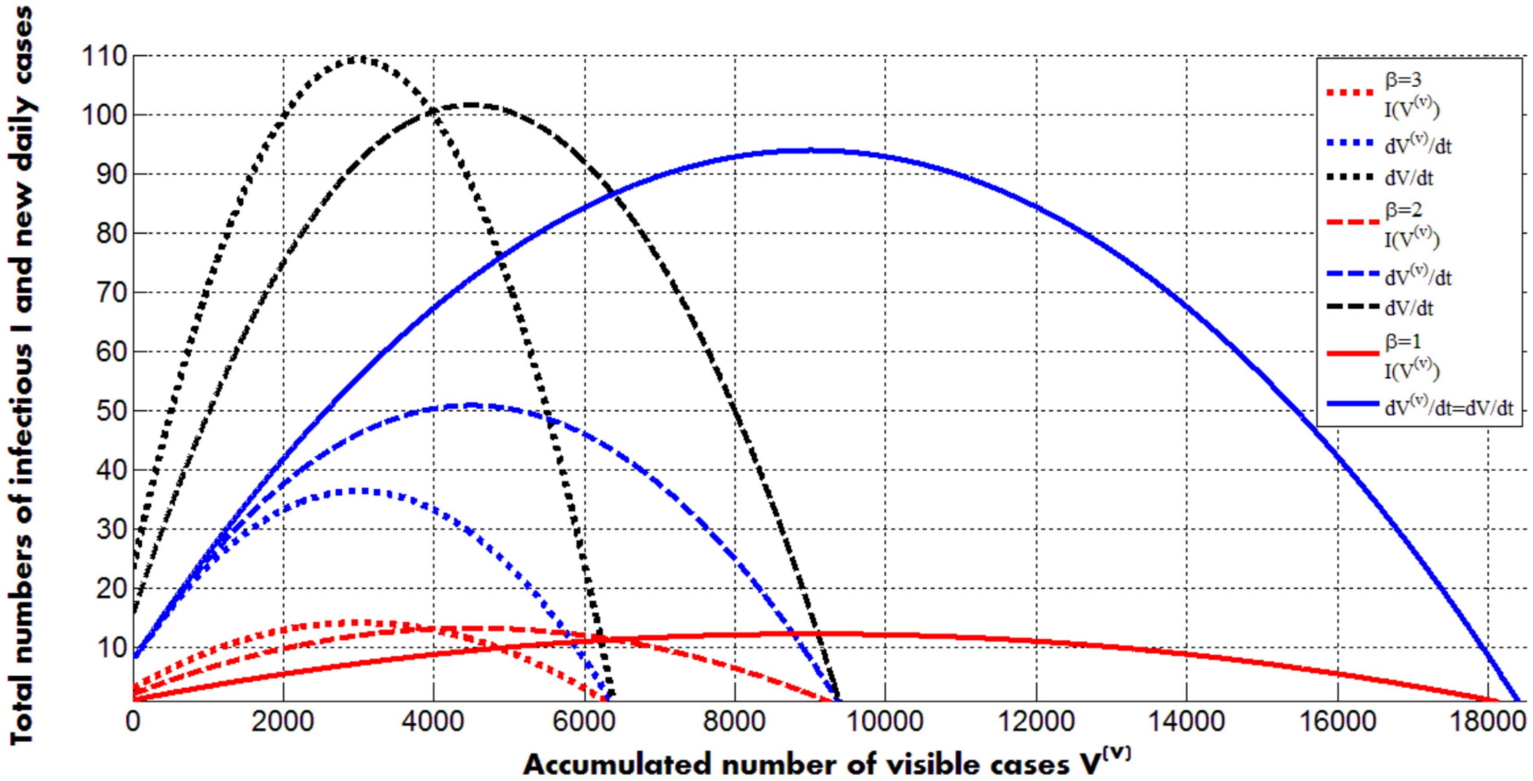
Solid, dashed and dotted curves represent calculations for values of the visibility coefficient 1; 2 and 3, respectively. Other values of parameters correspond to the second pertusis wave in England in 2023 and 2024 (listed in the previous Section). Red color corresponds to the real number of infectious persons (symptomatic and asymptomatic, Equation 28). Blue lines represent visible numbers of new daily cases; black ones - real (registered and hidden) numbers of new daily cases (Equations 28, 29).

Figure 3 represents the calculations of real number of infectious *I* (visible and hidden, Equation 28) and visible and real numbers of new daily cases (Equation 29) versus accumulated numbers of visible cases $V^{\left(v\right)}$ for different values of the visibility coefficient. Values of other parameters correspond to the second pertussis wave in England (listed in the previous Section).

The maximum values of infectious slightly increase with the increase of the visibility coefficient (compare red lines in Figure 3). The black and the solid blue curves show the same but more pronounced trend for the real numbers of new daily cases. The positions of the maxima on these lines are very close to ones on the red curves. Corresponding moments of time can be calculated with the use of Equations 19, 20, 24. Thus, the average daily numbers of visible cases reflects trends in real numbers of infectious persons (symptomatic and asymptomatic) and can be used to control epidemics. The numbers of new visible daily cases decrease with the increase of visibility coefficient (for fixed values of other parameters and $\rho_{i}^{\left(v\right)}=\rho_{i}^{\left(h\right)}=\rho_{i}$, see blue curves in Figure 3). The final numbers of visible cases demonstrate the same trend (see solid curves in Figure 2).

## 6 Conclusion

To simulate hidden epidemic dynamics connected with asymptomatic and unregistered patients, a new general SIR model was proposed containing 5 unknown functions. For some cases, the analytical solutions of the set of 5 differential equations were found which allow simplifying the parameter identification procedure. Two waves of the pertussis epidemic in England in 2023 and 2024 were simulated for the case of zero hidden cases. Observations of accumulated visible numbers of cases during 4 months revealed rather high accuracy of predictions. If trend will not change, the monthly figure of 9 new pertussis cases (as it was in January–February 2023) can be achieved only in May 2025. The proposed approach can be recommended both for preliminary simulations of different epidemics (supposing zero hidden cases) and for further research, using presented analytical solutions or numerical integration of differential equations. The theoretical estimations of numbers of hidden cases will allow healthcare professionals to know the real sizes of epidemics and to develop the right strategy without expensive mass testing.

## Data Availability

The original contributions presented in the study are included in the article/supplementary material, further inquiries can be directed to the corresponding author/s.
